# The role of ultrasound in the management and diagnosis of infectious mononucleosis

**DOI:** 10.1186/2036-7902-6-4

**Published:** 2014-02-28

**Authors:** Sarah N Farukhi, John C Fox

**Affiliations:** 1 University of California, Irvine School of Medicine, 101 The City Drive South, Orange, CA 92688, USA

**Keywords:** Ultrasound, Infectious mononucleosis, Splenomegaly

## Abstract

Currently, infectious mononucleosis (IM) is a clinically diagnosed condition. According to the American Family Physician criteria for IM, splenomegaly is the key factor that distinguishes IM from other causes of sore throat. Though heterophile antibody tests are often ordered to confirm diagnosis of IM, this test has a high false-negative rate early in the course of the disease. This case report provides an example of how the use of ultrasound to diagnose splenomegaly and subsequently mononucleosis increases diagnostic accuracy.

## Background

Bedside ultrasound is a fast, accessible, and cost-effective tool for clinical evaluation of patient symptoms. This case report investigates the use of ultrasound in the evaluation and diagnosis of splenomegaly in mononucleosis.

Typically, mononucleosis is a clinically diagnosed condition [1]. Symptoms that should arouse clinical suspicion of mononucleosis include sore throat, lymph node enlargement, fever, and tonsillar enlargement. Key physical exam findings include splenomegaly, hepatomegaly, pharyngeal inflammation, and palatal petechiae [1]. Heterophile antibody tests may also be used to confirm the diagnosis alongside clinical signs; however, false negative results are relatively common early in the course of the infection [1].

Evaluation of splenomegaly is imperative in the diagnosis and patient management of mononucleosis. Though splenic rupture is a rare complication of splenomegaly, it is a life-threatening one that patients must be made aware of. Patients with splenomegaly are therefore advised to avoid contact sports for 3 to 4 weeks until the splenomegaly has resolved [2].

Among pediatric patients with mononucleosis, up to 50% of patients will present with an enlarged spleen [3]. According to a study conducted by Marco et al., splenomegaly is most accurately diagnosed via ultrasonic measurements of spleen volume [4]. As demonstrated by this case study, ultrasound was a precise and cost-effective method to diagnose splenic enlargement in a patient with mononucleosis.

## Case presentation

Mr. F was a 16-year-old previously healthy male with a 4-day history of sore throat. He had a dry cough and nausea, but denied fever, voice changes, or sick contacts.

On initial presentation to the Emergency Department (ED), the patient’s vital signs were as follows: pulse 98 beats per minute (bpm), respiratory rate 18 cycles per minute (cpm), temp 36.7°C, and blood pressure 133/74 mmHg. Physical exam revealed bilateral tonsillar exudate and swelling (R > L). He had tender cervical lymph nodes. The uvula was midline. No palatal petechiae were noted. No splenomegaly was appreciated on physical exam.

Based on presenting symptoms, the patient received two points on the centor criteria scale (+tonsillar exudate, +cervical adenopathy) [5]. He therefore received a rapid strep test and a throat culture at his initial visit to the ED. Both tests were negative and the patient was discharged.

At a follow-up visit to the ED 2 days later, the patient complained of progressively worsening pain in his throat. He reported that the pain peaked to a 10/10 while swallowing. The patient could only tolerate a liquid diet and had significantly decreased his oral intake. He also reported nausea and intermittent dry cough. The patient denied chest pain, shortness of breath, vomiting, diarrhea, or abdominal pain.

On second presentation to the ED, the patient was now tachycardic (112 bpm) with blood pressure reduction to 111/64 mmHg. He was afebrile. Physical exam was largely unchanged from previous. Abdomen was soft and nontender. Again, no splenomegaly was appreciated on exam.

Given the negative strep throat culture from the patient’s prior visit, mononucleosis was high on the differential. Since splenic enlargement was not detected on physical exam, ultrasound imaging was performed to rule out splenomegaly.

A p21 transducer in the abdominal examination mode was used for measurement. The patient was placed in a supine position. The probe was placed posteriorly below 12th rib on the patient’s left side and angled anteriorly. The spleen was measured to be 17.8 cm in axial length (NL 11 to 13 cm).

Based on the patient’s significant splenomegaly and 4-day history of sore throat, the patient was given a presumptive diagnosis of mononucleosis. He was treated symptomatically: given IVF and anti-nausea and pain medications. After IVF, the patient’s HR decreased to 98 bpm. He was advised to avoid contact sports for the next 3 to 4 weeks. The patient was subsequently discharged with no further testing.

## Conclusion

Sore throat is a common chief complaint. However, the presence of palatal petechiae, splenomegaly, and posterior cervical adenopathy are highly suggestive of infectious mononucleosis [1]. As was seen in the case above, both physical examinations the patient received during his two ED visits were negative for splenomegaly. However, in cases where high suspicion for splenomegaly exists, further imaging should be obtained to rule it out. A recent study conducted by UCSD has shown wide variability in the ability to appreciate an enlarged spleen by physical exam; this finding was not directly correlated with the level of clinical experience [2]. In this scenario, the splenomegaly was a significant one (17.5 cm), and its lack of detection on physical exam skewed the patient’s clinical management.

The delayed diagnosis of mononucleosis had financial repercussions as well. On average, Medicare charges for a single visit to ED of sore throat or an upper respiratory tract infection are about $1,101 [4]. At his initial visit, this patient received a throat culture ($11.84) [6] and a strep A Ag test ($16.49) [6]. At his second ED visit, the patient had abdominal real time ultrasound imaging ($29.59) [5]. His entire diagnostic workup alongside his two ED visits totaled to about $2,260.

We hypothesize that an earlier ultrasound of the patient’s spleen would have led to an earlier diagnosis of mononucleosis. Subsequently, the patient could have been told what to expect from the course of this disease. In UptoDate’s ‘Patient information: infectious mononucleosis (mono) in adults and adolescents (Beyond the Basics)’, patients are reassured that symptoms of pain in mononucleosis can last from 2 to 4 weeks [7]. This reassurance along with adequate pain control measures may have prevented the second ED visit. The cost reduction in this case would have been close to $1,000.

Given that almost 50% to 60% of adolescents with mononucleosis have splenomegaly [1], the diagnosis of splenomegaly is a crucial step in management. Mr. F was a 16-year-old athletic male, highly involved in contact sports. At his initial visit, no splenomegaly was detected, so the patient was not told to avoid contact sports at discharge. Though splenic rupture is a rare complication of splenomegaly, the risk increases significantly in patients such as Mr. F who are actively involved in contact sports [8].

It is important to note that ultrasound holds potential not only in diagnosis of mononucleosis but also in monitoring the disease. Though most cases of splenomegaly resolve within 3 to 4 weeks, some do not [9]. In patients actively involved in contact sports, ascertaining that the spleen has regressed to normal size is crucial prior to allowing the patient to return to the sport.

## Consent

Phone consent was obtained from the patient for publication of this Case Report and any accompanying images. A document affirming this phone call is available for review by the Editor-in-Chief of the journal.

## Abbreviations

ED: Emergency Department; IM: infectious mononucleosis; IVF: intravenous fluid; L: left; Mono: mononucleosis; NL: normal; Strep: streptococcal; R: right.

## Competing interests

The authors declare that they have no competing interest.

## Authors’ contributions

SF performed the ultrasound scan, researched the cost-benefit analysis based on medicare prices, researched the association of mononucleosis and splenomegaly, and drafted the manuscript. CF supervised and reconfirmed the ultrasound scan, participated in the cost benefit analysis research, and helped draft the manuscript. Both authors read and approved the final manuscript.

## Authors’ information

SF is a fourth year medical student at the University of California, Irvine. She has received extensive training through the course of her medical school career in ultrasound technique. She has also participated in research studies analyzing the cost effectiveness of ultrasound in medical care. JF is an Emergency Medicine Physician at the University of California, Irvine Medical Center with fellowship training in Emergency Ultrasound. He is a world-renowned expert in the field of ultrasound technology with over 40 publications discussing the use of ultrasound in various aspects of clinical management. JF is a professor of ultrasound at UC Irvine School of Medicine as well as the Dean of Academic Affairs. He is a member of the American Institute of Ultrasound in Medicine.
